# Teleoperated Surgical Robot with Adaptive Interactive Control Architecture for Tissue Identification

**DOI:** 10.3390/bioengineering10101157

**Published:** 2023-10-02

**Authors:** Yubo Sheng, Haoyuan Cheng, Yiwei Wang, Huan Zhao, Han Ding

**Affiliations:** State Key Laboratory of Intelligent Manufacturing Equipment and Technology, Huazhong University of Science and Technology, Luoyu Road 1037, Wuhan 430074, China; sheng_yubo@hust.edu.cn (Y.S.); m202170692@hust.edu.cn (H.C.); huanzhao@hust.edu.cn (H.Z.); dinghan@hust.edu.cn (H.D.)

**Keywords:** teleoperated surgical robotics, adaptive control, haptic feedback, physical human–robot interaction

## Abstract

The remote perception of teleoperated surgical robotics has been a critical issue for surgeons in fulfilling their remote manipulation tasks. In this article, an adaptive teleoperation control framework is proposed. It provides a physical human–robot interaction interface to enhance the ability of the operator to intuitively perceive the material properties of remote objects. The recursive least square (RLS) is adopted to estimate the required human hand stiffness that the operator can achieve to compensate for the contact force. Based on the estimated stiffness, a force feedback controller is designed to avoid the induced motion and to convey the haptic information of the slave side. The passivity of the proposed teleoperation system is ensured by the virtual energy tank. A stable contact test validated that the proposed method achieved stable contact between the slave robot and the hard environment while ensuring the transparency of the force feedback. A series of human subject experiments was conducted to empirically verify that the proposed teleoperation framework can provide a more smooth, dexterous, and intuitive user experience with a more accurate perception of the mechanical property of the interacted material on the slave side, compared to the baseline method. After the experiment, the design idea about the force feedback controller of the bilateral teleoperation is discussed.

## 1. Introduction

Teleoperated robotics have attracted numerous interest in the recent decade. They provide a human–robot interface that enables operators to accomplish tasks by combining the human perception and the robot’s capabilities in two separate spaces [1,2]. Medical surgery is a typical application scenario for teleoperated robotics [3], as they provide surgeons with greater dexterity and more operational degrees of freedom [4,5]. A teleoperation surgical system contains two major parts: the master interface which collects the actions of the human operator, and the slave manipulator which reproduces the actions the master device collected. Surgeons are required to distinguish the stiffness of the tissue to find lesions (such as tumors or calcified arteries) from normal organs [6]. However, since the operators are separated from the environment, it is difficult for them to perceive the interactive information such as the contact force and tactile sensing. Therefore, it is necessary to research remote sensing methods for teleoperated surgery [7].

The primary way to achieve remote perception is by providing a haptic interface with a transparent implementation of the teleoperation system [8]. Transparency is defined as a correspondence between the master and slave positions (kinematic correspondence) and forces [9,10], or a match between the impedance perceived by the operator and the environment impedance [11]. The haptic interface conveys the information of the slave environment to the surgeon utilizing force feedback [8].

Impedance matching is a traditional method to achieve remote perception, which requires modeling the environment and estimating its mechanical properties. Many researchers adopted classical linear models such as the spring model [12] or the Kelvin–Voigt model [13] to describe the human tissue, although these approaches showed physical inconsistencies in terms of power exchange during the interaction [14]. Jeon et al. used the nonlinear Hunt–Crossley model, which takes into account the energy loss during impact observed in the linear tissue model [15]. Yamamoto et al. compared seven candidate models. They recommended the nonlinear Hunt–Crossley model for characterizing the tissue properties [6]. Based on the Hunt–Crossley model, force feedback controllers were designed to enhance the remote sensing for surgical teleoperation [16,17]. However, the neglected nonlinear terms may affect the linearized controller and perform negatively [17].

Direct force feedback is a straightforward way to convey interactive information, which simply recreates the force measured on the slave side without modeling the environment [18]. Recent research suggests that the position–force-based frameworks are capable of providing accurate remote perception in most cases [19,20,21]. However, when the slave robot contacts a hard object, the rapidly increasing feedback force will cause a violent recoiling of the master device and subsequently lead to the induced motion of the human operator [19], making the system’s contact with the environment discontinuous [22]. In this case, it is difficult for the surgeon to intuitively perceive the mechanical characteristics of the manipulated tissue. Furthermore, the induced motion causes the slave robot to move back and forth, which is dangerous for the patient. To avoid induced motion and maintain stable contact, several approaches have been extensively investigated. Kuchenbecker et al. enhanced the contact stability of the teleoperation system by canceling the induced motion on the master side [19]. However, this method is limited to linear systems. Polushin et al. presented a projection algorithm of force reflection to achieve stable contact [20,21]. However, this approach could distort the operator’s perception because of the anisotropic scaling of the environmental force. A conventional way to maintain the contact stability of the teleoperation system is to keep the system passive [23]. Ryu et al. developed the time domain passivity approach (TDPA) to ensure the passivity of the teleoperation system by monitoring the power flows through the energy ports [23,24]. However, the excess damping, related to the worst case, would distort the force perception conveyed to the operator. In recent years, a two-layer approach has been proposed [25]. It divided the teleoperation system into two layers—the transparency layer and the passivity layer—to achieve force feedback performance and passivity, respectively. In the passivity layer, energy tanks were used to guarantee the passivity of the teleoperation system by monitoring the energy stored in the tank [26]. This method has attracted much research interest [18,27,28]. However, when the teleoperation system is not passive, the connection between the master and the slave subsystems will be interrupted, which makes the teleoperation control discontinuous.

Since teleoperated robots are typically human-in-the-loop systems, human behaviors can be utilized to enhance the performance of teleoperation. An intention prediction method using the human hand motion was proposed to improve the tracking performance of the slave robot in [29]. Qi et al. presented a touch-free guided hand gesture recognition system for surgical robot teleoperation [30]. A framework of human-ware control to achieve a safe and compliant motion interaction for a teleoperated wheelchair system was designed in assistive task [31]. However, to the best of our knowledge, few studies use the adaptive behavior of humans on the master side to enhance remote sensing in real-time.

This study focuses on a scenario where surgeons can intuitively perceive the characteristics of manipulated objects when the slave robot probes different tissues. To achieve this goal, an adaptive teleoperation control scheme based on the adaptability of the human hand is proposed. As described in [20], the perceptible force, through which the operator obtains information about the remote environment, is the key component of the feedback force compensated by the human hand. The stiffness of the human hand is required to increase for compensation. However, most of the operators are incapable of adjusting their hand stiffness quickly enough to fully compensate for the feedback force during the interaction, which eventually leads to induced motion. Therefore, a gradual force feedback control scheme is proposed, which is obedient to the adaptability exhibited by the operator when interacting directly with the real environment. The recursive least square (RLS) is adopted to estimate the required human hand stiffness that the operator can achieve. A feedback force controller is designed, which avoids the induced motion and conveys the haptic information of the slave side at the same time. Then, an energy tank mechanism is adopted to ensure the passivity of the teleoperation system [32]. Human subject experiments are conducted to verify the feasibility of the proposed method.

The main contributions of this paper can be summarized as follows. An adaptive stiffness force feedback controller was presented by estimating the required human hand stiffness to convey the haptic information to the master side without tissue models. A teleoperation framework ensuring contact stability and passivity was constructed. Human subject experiments were conducted to verify that the proposed method accords with human intuition when the slave robot contacts unknown objects with different mechanical properties. A force feedback mechanism of bilateral teleoperation was discovered in the process of testing the method.

The rest of this article is organized as follows. Section 2 introduces the proposed method and analyzes the passivity. The experimental results are given in Section 3. Section 4 discusses the finding of this study. Section 5 presents the conclusion.

## 2. Methods

**Figure 1:**
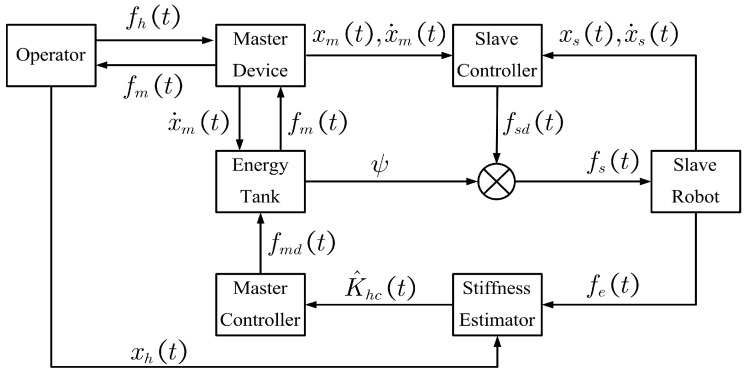
The block diagram summarizes the developed teleoperation framework. The estimated stiffness ${\hat{K}}_{hc}\left(t\right)$ is obtained according to the human hand motion and the contact force between the slave robot and the environment. The master controller realizes the adaptive force feedback control based on the estimated stiffness. The energy tank ensures the passivity of the teleoperation system by modifying the feedback force.

In this section, the proposed adaptive control method for the discussed scenario is introduced. The overall design of the developed control framework is described in Figure 1. It can be seen that the required stiffness is estimated by utilizing the human hand motion and the contact force between the slave robot and the environment. The feedback is adapted to the estimated stiffness. Furthermore, the energy tank modifies the feedback force to ensure the passivity of the whole system. The symbols appearing in the figure are all explained in the context.

### 2.1. Teleoperation Bilateral Contact Model

**Figure 2:**
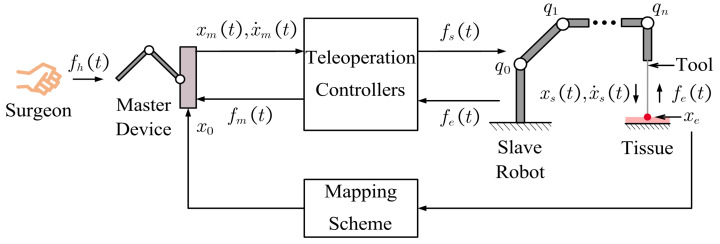
The interactive model depicts the process where the surgeon probes the tissue through the teleoperated robot. When the slave robot interacts with the tissue at contact position $x_{e}$, the surgeon perceives the feedback force at virtual contact position $x_{0}$.

The discussed scenario is depicted in Figure 2. It can be seen that the surgeon on the master side operates the master device to control the slave robot to probe the remote tissue on the slave side. A tool attached to the end of the slave robot is used to interact with the environment, whereas the master device plays the role of a haptic interface to convey the tissue stiffness to the surgeon. The subscript *m* stands for the master and the subscript *s* for the slave, both of which can be denoted as $i\in \left\{m,s\right\}$. $x_{i}\left(t\right)\in R^{3}$ and ${\dot{x}}_{i}\left(t\right)\in R^{3}$ are position and velocity, respectively. $f_{h}\left(t\right)\in R^{3}$ and $f_{e}\left(t\right)\in R^{3}$ are the master interaction force and the slave contact force. Teleoperation controllers produce the control commands based on $x_{m}\left(t\right),{\dot{x}}_{m}\left(t\right)$ from the master device and $f_{e}\left(t\right)$ from the slave robot. $f_{m}\left(t\right)\in R^{3}$ and $f_{s}\left(t\right)\in R^{3}$ are the control commands of the master and the slave used to generate the feedback force and to activate the remote robot, respectively. $x_{e}\in R^{3}$ is the contact position when the slave robot interacts with the environment. When the slave robot moves to $x_{e}$, the contact force is generated and the operator perceives the feedback force on the master side as if interacting with an unknown object. Therefore, there is a virtual contact position corresponding to the contact position on the master side, denoted as $x_{0}\in R^{3}$. The relationship between $x_{0}$ and $x_{e}$ can be expressed by the teleoperation mapping scheme between the master and slave sides.

The dynamic models of the master and the slave manipulators in the Cartesian space can be expressed as [33]

(1) $$\left\{\begin{array}{c} M_{m}\left(x_{m}\left(t\right)\right){\ddot{x}}_{m}\left(t\right)+C_{m}\left(x_{m}\left(t\right),{\dot{x}}_{m}\left(t\right)\right){\dot{x}}_{m}\left(t\right)\\ \;=f_{h}\left(t\right)+f_{m}\left(t\right)\\ M_{s}\left(x_{s}\left(t\right)\right){\ddot{x}}_{s}\left(t\right)+C_{s}\left(x_{s}\left(t\right),{\dot{x}}_{s}\left(t\right)\right){\dot{x}}_{s}\left(t\right)\\ \;=f_{s}\left(t\right)-f_{e}\left(t\right) \end{array}\right.$$

where $M_{i}\left(x_{i}\left(t\right)\right)\in R^{3\times 3}$ denotes the positive definite and symmetric inertia matrix. The matrix $C_{i}\left(x_{i}\left(t\right),{\dot{x}}_{i}\left(t\right)\right)\in R^{3\times 3}$ represents the Coriolis and Centrifugal effects. Conventionally, the matrix $\left({\dot{M}}_{i}\left(x_{i}\left(t\right)\right)-2C_{i}\left(x_{i}\left(t\right),{\dot{x}}_{i}\left(t\right)\right)\right)$ is skew symmetry [32].

### 2.2. Teleoperation Mapping Scheme

The teleoperation scheme adopts an incremental Cartesian position mapping between master and slave sides in three-dimensional coordinates through a switch mechanism similar to [18] to compensate for the operable workspace differences.

When the teleoperation is switched on, the bilateral control is activated. The slave robot is guided by the master manipulator incrementally:

(2) $$\left\{\begin{array}{c} x_{sd}=x_{son}+\xi \int_{\delta_{on}}^{\delta_{curr}}R_{m}^{s}{\dot{x}}_{m}\left(\delta \right)d\delta \\ x_{md}=x_{hd} \end{array}\right.$$

where $R_{m}^{s}\in R^{3\times 3}$ is a rotation matrix from the slave manipulator to the master. $x_{sd}\in R^{3}$ and $x_{md}\in R^{3}$ are the desired Cartesian positions of the slave robot and the master device, respectively. $x_{hd}\in R^{3}$ is the desired Cartesian position of the operator. In the discussed case, $x_{md}$ is set to follow $x_{hd}$. The position $x_{son}\in R^{3}$ is the instant slave Cartesian position at the moment $\delta_{on}$ when the connection is on. The coefficient, ξ∈R, is a positive scaling rate for mapping. Furthermore, $\delta_{curr}$ is the current time.

When the teleoperation is switched off, the bilateral control is given as follows

(3) $$\left\{\begin{array}{c} x_{sd}=x_{soff}\\ x_{md}=x_{hd} \end{array}\right.$$

where $x_{soff}\in R^{3}$ is the instant slave Cartesian position at the moment $\delta_{off}$. The slave robot remains stationary, and there is no force feedback from the master manipulator. Furthermore, the master device can be moved freely according to the operator.

Based on the adopted teleoperation mapping scheme, the relation between $x_{0}$ and $x_{e}$ can be specified as

(4) $$x_{0}=x_{mon}+x_{e}-x_{son}$$

where $x_{mon}\in R^{3}$ is the instant master Cartesian position at the moment $\delta_{on}$ when the teleoperation is switched on.

### 2.3. Interaction Force Model

The properties of the interaction force in the process of remote probing are discussed. The surgeon needs to operate the master device and compensate for the unexpected feedback force. Therefore, the interaction force between the surgeon’s hand and the master device is divided into two parts as follows

(5) $$f_{h}\left(t\right)=f_{hd}\left(t\right)+f_{hc}\left(t\right)$$

where $f_{hd}\left(t\right)\in R^{3}$ is the force produced by the active action of the operator that can guide the master device to the desired position. Furthermore, $f_{hc}\left(t\right)\in R^{3}$ is used to compensate for the unexpected feedback force generated when the slave robot interacts with the environment.

When no feedback force is produced, $f_{h}\left(t\right)=f_{hd}\left(t\right)$. Furthermore, $f_{hd}\left(t\right)$ can be expressed as follows [34]

(6) $$f_{hd}\left(t\right)=K_{0}\left(x_{h}\left(t\right)-x_{hd}\right)$$

where $x_{h}\left(t\right)\in R^{3}$ is the current position of the operator and $K_{0}\in R^{3\times 3}$ is the diagonal stiffness matrix. Since the resistance is low without the feedback force, $f_{hd}\left(t\right)$ is typically very small.

When the slave robot contacts the environment, the operator perceives the abrupt change in feedback force at the virtual contact position $x_{0}$. The interaction force needs to be increased for the compensation by providing additional stiffness of the human hand, which means $f_{hc}\left(t\right)$ can be expressed similarly to Equation (6).

(7) $$f_{hc}\left(t\right)=K_{h}\left(t\right)\left(x_{h}\left(t\right)-x_{0}\right)$$

where $K_{h}\left(t\right)\in R^{3\times 3}$ is the diagonal stiffness matrix increased for the compensation. To achieve stable contact, the additional interaction force needs to fully compensate for the feedback force in real-time, which implies $f_{hc}\left(t\right)=-f_{m}\left(t\right)$.

### 2.4. The Ideal Interaction Situation

We note that the human–environment interaction is always stable when the surgeon probes the tissue directly. At this point, the interaction force exerted by the human can be written as

(8) $$f_{hc}^{*}\left(t\right)=K_{hc}\left(t\right)\left(x_{h}\left(t\right)-x_{0}\right)$$

In this case, the virtual contact position is the same as the environment position $x_{e}$. $K_{hc}\in R^{3\times 3}$ is the hand stiffness achieved by the human and can be adaptively adjusted according to the object being interacted with. During direct probing, the equation $f_{e}\left(t\right)=f_{hc}^{*}\left(t\right)$ always holds, which means that the contact force will gradually increase in compliance with the interaction force and can be fully compensated by human hands.

During the teleoperation depicted in Section 2.1, the operator perceives the environment by compensating for the feedback force through the interaction force $f_{hc}\left(t\right)$ at the virtual contact position $x_{0}$. The haptic interface needs to completely convey the contact force on the slave side for transparency, i.e., $f_{m}\left(t\right)=-f_{e}\left(t\right)$. To achieve accurate remote perception and stable contact, the surgeon is expected to fully compensate for the feedback force. However, the rapidly increasing feedback force prevents the operator from adjusting the arm stiffness in time and eventually leads to induced motion. The interaction would be stable if the feedback force from the virtual contact position could be conveyed to the surgeon gradually according to the stiffness of the human hand, as in the case of direct interaction between the operator and the real environment. Since Equation (8) describes the interaction force characteristic during direct contact, $f_{hc}^{*}\left(t\right)$ is taken as the ideal case for $f_{hc}\left(t\right)$, and $K_{hc}\left(t\right)$ denotes the required stiffness that the operator needs to achieve during the teleoperation. Thus, the feedback force should increase gradually according to the required human hand stiffness as if directly probing. A stiffness estimator is introduced to estimate the required hand stiffness. The feedback force controller is designed to replicate the adaptability of the human in the master device to avoid the induced motion and enhance the surgeon’s ability to identify different tissues.

### 2.5. Stiffness Estimation

As discussed in Section 2.4, to ensure stability, the interaction of the operator with the feedback force at the virtual contact position $x_{0}$ is expected to be the same as the direct contact with the environment, which implies

(9) $$f_{e}\left(t\right)=K_{hc}\left(t\right)\left(x_{h}\left(t\right)-x_{0}\right)$$

The contact force $f_{e}\left(t\right)$ can be measured by the force sensor.

Subscript $j\in \left\{x,y,z\right\}$ represents the component along the coordinate axis in Cartesian space. Therefore, $f_{e,j}\left(t\right)\in R$ and $x_{h,j}\left(t\right)\in R$ are the components of the contact force and the human hand position, respectively. $k_{hc,j}\left(t\right)\in R^{+}$ is the diagonal element of $K_{hc}\left(t\right)$ representing the required stiffness in each axis. Denote $x_{h,j}\left(t\right)-x_{0,j}={\tilde{x}}_{j}\left(t\right)$, where $x_{0,j}\in R$ is the component of the virtual contact position. The scaling rate is set as ξ=1. Then, an RLS-based estimator which is similar to [35] is used to obtain the required estimated stiffness ${\hat{k}}_{hc,j}\left(t\right)$. Before introducing the estimator, the following assumption is necessary to guarantee the performance of the designed controller.

**Assumption** **1.** *During the teleoperated probing, the required increased stiffness $K_{hc}\left(t\right)$ tends to be constant.*

The performance index can be expressed by

(10) $$f_{e,j}\left(t\right)={\tilde{x}}_{j}^{T}\left(t\right)k_{hc,j}\left(t\right)$$

The estimator according to the sampling time *t* is given as follows:

(11) $${\hat{f}}_{e,j}\left(t\right)={\tilde{x}}_{j}^{T}\left(t\right){\hat{k}}_{hc,j}\left(t-1\right)$$

(12) $$P_{j}\left(t\right)=\frac{1}{\lambda }\left(P_{j}\left(t-1\right)-\frac{P_{j}\left(t-1\right){\tilde{x}}_{j}\left(t\right){\tilde{x}}_{j}^{T}\left(t\right)P_{j}\left(t-1\right)}{\lambda +{\tilde{x}}_{j}^{T}\left(t\right)P_{j}\left(t-1\right){\tilde{x}}_{j}\left(t\right)}\right)$$

(13) $${\hat{k}}_{hc,j}\left(t\right)={\hat{k}}_{hc,j}\left(t-1\right)+P_{j}\left(t\right){\tilde{x}}_{j}\left(t\right)\left(f_{e,j}\left(t\right)-{\hat{f}}_{e,j}\left(t\right)\right)$$

where ${\hat{f}}_{e,j}\left(t\right)$ is the current estimated value of the compensated force calculated by the previously estimated stiffness ${\hat{k}}_{hc,j}\left(t-1\right)$, the constant λ∈(0,1] is the forgetting factor and $P_{j}\left(t\right)\in R^{+}$ is the inverse of the weighted sample covariance matrix whose initial value is positive definite. Then, the overall estimated stiffness ${\hat{K}}_{hc}\left(t\right)$ can be obtained.

### 2.6. Desired Teleoperation Controllers

The desired master controller (force feedback controller) is designed by learning the stiffness variation characteristic of the human hand as the following

(14) $$f_{md}\left(t\right)=-{\hat{K}}_{hc}\left(t\right)\left(x_{m}\left(t\right)-x_{0}\right)$$

Equation (14) indicates that the feedback force is applied to the operators based on their adaptive behaviors. The feedback force increases gradually in obedience to the motion of the human hand which does not abruptly change. Therefore, operators are capable of adjusting their hand stiffness to avoid the induced motion, which achieves a stable and safe interaction between the slave robot and the slave environment. The estimated value ${\hat{K}}_{hc}\left(t\right)$ can be regarded as the adaptability of the human hand stiffness when probing different objects directly. The proposed force feedback controller replicates the adaptability to convey the haptic information of the slave environment to the operator indirectly.

The feedback force generated by the controller needs to eventually converge to the actual contact force for transparency. Then, the following theorem holds.

**Theorem** **1.** *If Assumption 1 is satisfied, the transparency of the force feedback controller can be ensured, which means that equation $\lim_{t\to \infty }∥f_{md}\left(t\right)+f_{e}\left(t\right)∥=0$ holds.*

**Proof of Theorem 1.**  Since the operator always holds the master device, the position of the operator can be regarded as consistent with the position of the master manipulator, which means $x_{h}\left(t\right)=x_{m}\left(t\right)$. Furthermore, Equation (14) can be rewritten as

(15) $$f_{md}\left(t\right)=-{\hat{K}}_{hc}\left(t\right)\left(x_{h}\left(t\right)-x_{0}\right)$$

Let $f_{md,j}\left(t\right)\in R$ represent the component of the feedback force. It can be seen that $f_{md,j}\left(t\right)=-{\tilde{x}}_{j}^{T}\left(t\right){\hat{k}}_{hc,j}\left(t\right)$. Furthermore, (13) can be equivalently stated as

(16) $$\begin{array}{c} {\hat{k}}_{hc,j}\left(t\right)-{\hat{k}}_{hc,j}\left(t-1\right)=\frac{P_{j}\left(t-1\right){\tilde{x}}_{j}\left(t\right){\tilde{x}}_{j}^{T}\left(t\right)}{\lambda +{\tilde{x}}_{j}^{T}\left(t\right)P_{j}\left(t-1\right){\tilde{x}}_{j}\left(t\right)}\left(k_{hc,j}\left(t\right)-{\hat{k}}_{hc,j}\left(t-1\right)\right) \end{array}$$

The tracking error between the estimated stiffness and the actual value can be defined as

(17) $$\Delta k_{hc,j}\left(t\right)={\hat{k}}_{hc,j}\left(t\right)-k_{hc,j}\left(t\right)$$

and then

(18) $$\Delta k_{hc,j}\left(t\right)=\left(I_{j}-\frac{P_{j}\left(t-1\right){\tilde{x}}_{j}\left(t\right){\tilde{x}}_{j}^{T}\left(t\right)}{\lambda +{\tilde{x}}_{j}^{T}\left(t\right)P_{j}\left(t-1\right){\tilde{x}}_{j}\left(t\right)}\right)\Delta k_{hc,j}\left(t-1\right)$$

where $I_{j}\in R$ is an identity matrix.

Since the following equations hold using the Euclidian 2-norm

(19) $$\begin{array}{c} ||I_{j}-\frac{P_{j}\left(t-1\right){\tilde{x}}_{j}\left(t\right){\tilde{x}}_{j}^{T}\left(t\right)}{\lambda +{\tilde{x}}_{j}^{T}\left(t\right)P_{j}\left(t-1\right){\tilde{x}}_{j}\left(t\right)}||=1-\frac{∥{\tilde{x}}_{j}^{T}\left(t\right)P_{j}\left(t-1\right){\tilde{x}}_{j}\left(t\right)∥}{\lambda +{\tilde{x}}_{j}^{T}\left(t\right)P_{j}\left(t-1\right){\tilde{x}}_{j}\left(t\right)}<1 \end{array}$$

Then combining (18) and (19) yields

(20) $$\lim_{t\to \infty }∥{\hat{k}}_{hc,j}\left(t\right)-k_{hc,j}\left(t\right)∥=0$$

Then, the following equation can be gained

(21) $$\lim_{t\to \infty }∥f_{md,j}\left(t\right)+f_{e,j}\left(t\right)∥=0$$

Equation (21) can be represented as follows by combining all the components.

(22) $$\lim_{t\to \infty }∥f_{md}\left(t\right)+f_{e}\left(t\right)∥=0$$

Consequently, Theorem 1 has been thoroughly proven. □

As for the slave robot, the control command is designed as a PD controller, such that the position of the robot end-effector follows the desired position

(23) $$f_{sd}\left(t\right)=K_{p}\left(x_{sd}-x_{s}\left(t\right)\right)+K_{d}\left({\dot{x}}_{sd}-{\dot{x}}_{s}\left(t\right)\right)$$

where $K_{p}\in R^{3\times 3}>0$ and $K_{d}\in R^{3\times 3}>0$ are the proportional and the derivative gains, respectively.

### 2.7. Passivity Analysis

Ensuring safety is a priority goal for the teleoperated surgical robot system. As described in Section 2.6, the feedback force is generated gradually, which avoids the induced motion. Therefore, the interaction between the slave robot and the patient can be safe through the designed force feedback controller. Furthermore, the teleoperation system is required to preserve passivity to guarantee additional safety and robustness during interaction with unknown environments [36].

The passivity needs to be maintained with respect to the ports $\left(f_{h}\left(t\right),{\dot{x}}_{m}\left(t\right)\right)$ and $\left(-f_{e}\left(t\right),{\dot{x}}_{s}\left(t\right)\right)$, through which the system can interact with the external environments during the teleoperation [26]. Let W(t) denote the total energy of the system, $W_{m}\left(t\right)$ denote the energy of the master side, and $W_{s}\left(t\right)$ denote the energy of the slave side. The global storage function of the system can be expressed as

(24) $$\begin{array}{cc} W(t)= & W_{m}\left(t\right)+W_{s}\left(t\right)\\ = & \frac{1}{2}{\dot{x}}_{m}^{T}\left(t\right)M_{m}{\dot{x}}_{m}\left(t\right)+\frac{1}{2}{\dot{x}}_{s}^{T}\left(t\right)M_{s}{\dot{x}}_{s}\left(t\right)\\ \; & +\frac{1}{2}{\left(x_{m}\left(t\right)-x_{0}\right)}^{T}{\hat{K}}_{hc}\left(t\right)\left(x_{m}\left(t\right)-x_{0}\right)\\ \; & +\frac{1}{2}{\left(x_{s}\left(t\right)-x_{sd}\right)}^{T}K_{p}\left(x_{s}\left(t\right)-x_{sd}\right) \end{array}$$

Thus, the derivative of (24) can be represented as

(25) $$\begin{array}{cc} \dot{W}\left(t\right)= & {\dot{x}}_{m}^{T}\left(t\right)f_{h}\left(t\right)-{\dot{x}}_{s}^{T}\left(t\right)f_{e}\left(t\right)-{\dot{x}}_{s}^{T}\left(t\right)K_{d}{\dot{x}}_{s}\left(t\right)\\ \; & +\frac{1}{2}{\left(x_{m}\left(t\right)-x_{0}\right)}^{T}{\dot{\hat{K}}}_{hc}\left(t\right)\left(x_{m}\left(t\right)-x_{0}\right) \end{array}$$

Passivity implies that

(26) $$W\left(t\right)\leq {\dot{x}}_{m}^{T}\left(t\right)f_{h}\left(t\right)-{\dot{x}}_{s}^{T}\left(t\right)f_{e}\left(t\right)$$

however, the extra term $\frac{1}{2}{\left(x_{m}\left(t\right)-x_{0}\right)}^{T}{\dot{\hat{K}}}_{hc}\left(t\right)\left(x_{m}\left(t\right)-x_{0}\right)$ due to the variable stiffness of master device can be positive. Thus, inequality (26) cannot be satisfied strictly. To make the system maintain passive, the master subsystem is augmented with an energy tank [32,36] that can store the dissipated energy and modify the control command according to the amount of tank energy. The tank state model is defined as

(27) $${\dot{x}}_{t}\left(t\right)=\frac{\sigma }{x_{t}\left(t\right)}{\dot{x}}_{m}^{T}\left(t\right)D_{m}{\dot{x}}_{m}\left(t\right)-\frac{1}{x_{t}\left(t\right)}{\dot{x}}_{m}^{T}\left(t\right)f_{md}\left(t\right)$$

where $x_{t}\in R$ is the state of the tank and the storage function of the tank can be written as $W_{t}\left(t\right)=\frac{1}{2}x_{t}^{2}\left(t\right)$. Based on Equation (27), the energy tank can store the energy dissipated by the damping term $D_{m}\in R^{3\times 3}$, which can be set through damping injection into the master controller. Consequently, the master controller is modified as

(28) $$f_{m}\left(t\right)=f_{md}\left(t\right)-D_{m}{\dot{x}}_{m}\left(t\right)$$

**Figure 3:**
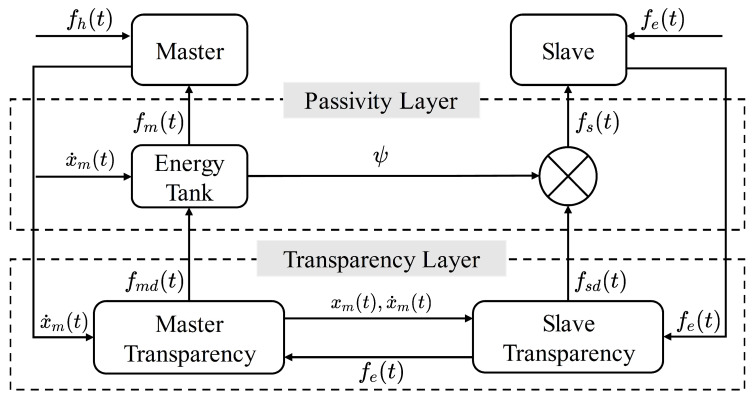
A two-layer control framework is constructed. The desired control commands are generated in the transparency layer. The energy tank in the passivity layer can modify the desired control commands according to the stored energy level $W_{t}\left(t\right)$ to ensure passivity.

A two-layer teleoperation control framework equipped with an energy tank is constructed, as shown in Figure 3. In the transparency layer, the master and slave exchange their positions, velocities, and force information to compute the desired controller. Then, the controllers are modified in the passivity layer through the energy tank. Equation (27) indicates that the tank can track the energy change caused by the desired controller $f_{md}\left(t\right)$. The tank then modifies the controllers according to the stored energy level. When the tank is empty, the system will not be passive anymore and the control of the system will be inhibited. Therefore, the master controller can be summarized as

(29) $$f_{m}\left(t\right)=\left\{\begin{array}{c} f_{md}\left(t\right)-D_{m}{\dot{x}}_{m}\left(t\right),\;W_{t}\left(t\right)>\varepsilon \\ 0,\;otherwise \end{array}\right.$$

where ε>0 is an arbitrarily small threshold to avoid singularities in (27).

The slave controller can be designed as

(30) $$f_{s}\left(t\right)=\left\{\begin{array}{c} f_{sd}\left(t\right),\;\psi =1\\ 0,\;\psi =0 \end{array}\right.$$

where ψ is a trigger signal depending on $W_{t}\left(t\right)$. When the tank energy is empty, the communication between the master and the slave will be interrupted to ensure safety. So ψ is defined as

(31) $$\psi =\left\{\begin{array}{c} 1,\;W_{t}\left(t\right)>\varepsilon \\ 0,\;otherwise \end{array}\right.$$

Furthermore, the tank energy should be properly bounded to avoid practically unstable behavior. Consequently, σ is set to prevent energy from becoming too large as follows

(32) $$\sigma =\left\{\begin{array}{c} 1,\;W_{t}\left(t\right)\leq \bar{T}\\ 0,\;otherwise \end{array}\right.$$

where $\bar{T}>0$ is a suitable application-dependent upper bound on the energy that can be stored in the tank.

Combining Equations (1) and (28), the dynamic model of the master subsystem can be rewritten as

(33) $$\begin{array}{cc} M_{m} & \left(x_{m}\left(t\right)\right){\ddot{x}}_{m}\left(t\right)+C_{m}\left(x_{m}\left(t\right),{\dot{x}}_{m}\left(t\right)\right){\dot{x}}_{m}\left(t\right)\\ \; & +D_{m}{\dot{x}}_{m}\left(t\right)=f_{h}\left(t\right)+f_{md}\left(t\right) \end{array}$$

Then, the passivity of the overall teleoperation system can be analyzed. The storage function (24) can be changed into

(34) $$W\left(t\right)=W_{m}\left(t\right)+W_{s}\left(t\right)+W_{t}\left(t\right)$$

Using (27), the derivative of Equation (34) can be obtained

(35) $$\begin{array}{cc} \dot{W}\left(t\right)= & {\dot{x}}_{m}^{T}\left(t\right)f_{h}\left(t\right)-{\dot{x}}_{s}^{T}\left(t\right)f_{e}\left(t\right)-{\dot{x}}_{s}^{T}\left(t\right)K_{d}{\dot{x}}_{s}\left(t\right)\\ \; & -{\dot{x}}_{m}^{T}\left(t\right)D_{m}{\dot{x}}_{m}\left(t\right)+{\dot{x}}_{m}^{T}\left(t\right)f_{md}\left(t\right)\\ \; & +\sigma {\dot{x}}_{m}^{T}\left(t\right)D_{m}{\dot{x}}_{m}\left(t\right)-{\dot{x}}_{m}^{T}\left(t\right)f_{md}\left(t\right)\\ = & {\dot{x}}_{m}^{T}\left(t\right)f_{h}\left(t\right)-{\dot{x}}_{s}^{T}\left(t\right)f_{e}\left(t\right)-{\dot{x}}_{s}^{T}\left(t\right)K_{d}{\dot{x}}_{s}\left(t\right)\\ \; & -\left(1-\sigma \right){\dot{x}}_{m}^{T}\left(t\right)D_{m}{\dot{x}}_{m}\left(t\right) \end{array}$$

Since $\sigma \in \left\{0,1\right\}$, the inequality (26) holds, therefore maintaining the passivity of the system.

## 3. Experiments and Results

To validate the proposed adaptive teleoperation framework, IRB-approved human subject experiments have been conducted, which will be discussed in this section.

### 3.1. Hardware Setup

**Figure 4:**
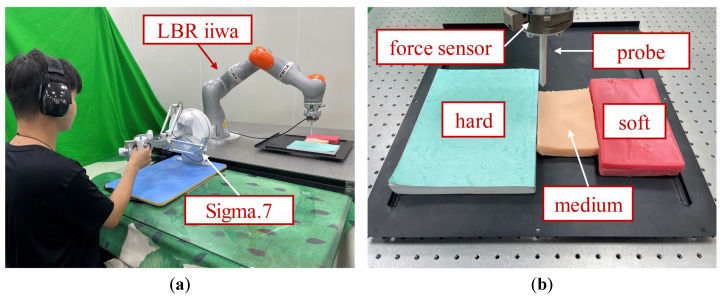
The experiment scenes are demonstrated: (**a**) Setup for the experiments. (**b**) Target objects with different stiffness. During the experiment, the green curtain was unfolded to block the sight of the participants.

The structure of the experiment setup is shown in Figure 4. A Force Dimension Sigma.7 haptic device was used as the master manipulator, and a KUKA LBR iiwa manipulator served as the slave robot. The contact force was measured by an ATI six-axis force/torque sensor. A metal probe was mounted on the end-effector of the slave robot to interact with the remote environment. The teleoperation system was implemented with the Robot Operating System (ROS). During the experiment, the forgetting factor in (12) and the damping injected into the master device in (28) were, respectively, set as λ=0.9 and $D_{m}=5$ Ns/m. The upper bound of the energy tank was set as $\bar{T}=20$ J to maintain passivity.

### 3.2. Stable Contact Test

Prior to the human subject experiments, a pilot test focusing on verifying the ability to maintain stable contact was conducted, where the slave robot was carefully manipulated to contact a rigid metal plate continuously with the proposed method and the direct force feedback approach, i.e., $f_{m}\left(t\right)=-f_{e}\left(t\right)$.

#### 3.2.1. Hypotheses

**Hypothesis** **1.** *The slave robot can maintain constant contact with the environment even if the feedback force becomes significant based on the developed teleoperation framework.*

**Hypothesis** **2.** *The feedback force generated by the proposed method can track the actual contact force well to ensure transparency.*

**Hypothesis** **3.** *The RLS-based stiffness estimator can quickly converge to the required human hand stiffness.*

#### 3.2.2. Results

**Figure 5:**
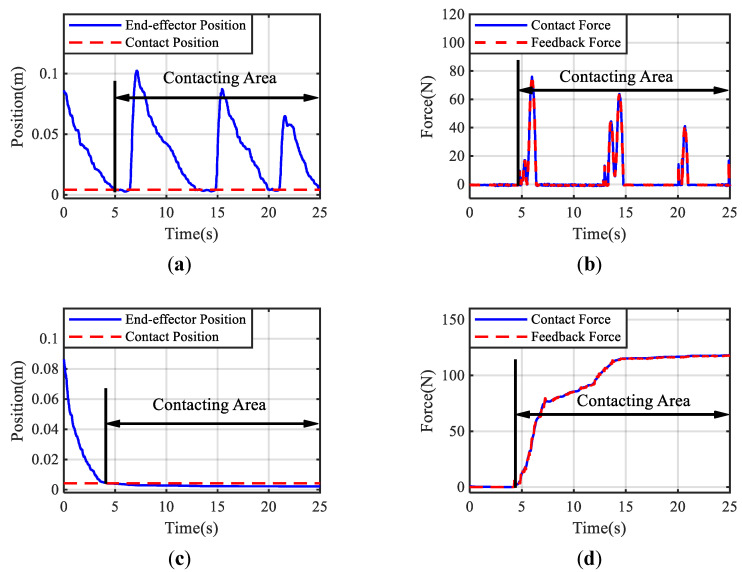
Responses of the teleoperated system when contacting the hard environment are shown in the figures. (**a**) The position of the end-effector tip in case of direct force feedback. It oscillates after contacting the environment at t≈4.6 s. (**b**) Transparency of the feedback force in case of direct force feedback. The feedback force increases rapidly by tracking the contact force. (**c**) The position of the end-effector tip in the case of the proposed method. It remains in the contact position after contacting the environment at t≈4.2 s. (**d**) Transparency of the feedback force in the case of the proposed method. The feedback force tracks the contact force well.

**Figure 6:**
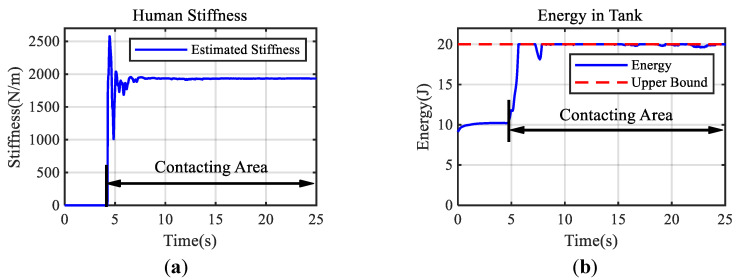
Response of the required estimated human hand stiffness and the energy stored in the tank are plotted. (**a**) The estimated human hand stiffness to generate the feedback force. It can be seen that the estimated stiffness converges to the required value quickly. (**b**) The energy stored in the tank.

Figure 5 demonstrates the positions of the slave manipulator end-effector and the interaction force under adaptive control and direct force feedback control. A series of rough and sluggish motions can be seen in Figure 5a, indicating that the rapidly increasing feedback force shown in Figure 5b caused the induced motion in the contact area with the direct force feedback method. In contrast, Figure 5c shows that the probing motion is continuous and smooth using the proposed approach (supporting Hypothesis 1). Comparing the contact force and feedback force shown in Figure 5d, it is promising that the feedback force under the designed teleoperation framework can track the contact force well to ensure transparency without violating the limits of human hand stiffness adaptation, even the maximum feedback force is larger than the one under the case of direct feedback force method (supporting Hypothesis 2). The performance of the proposed method for haptic teleoperation is demonstrated by the experimental results shown in Figure 6. The estimated stiffness for generating the feedback force is shown in Figure 6a. In the beginning, the estimated stiffness changed rapidly to balance the stability and transparency of the contact and then maintained at a constant level, which describes how the proposed approach stabilized the interaction to achieve the smooth probing task by considering the stiffness adaptation of the human hand. It took about 2.12 s for the estimated stiffness to converge to the required value (supporting Hypothesis 3). The energy stored in the tank configured on the master side and the upper bound of the energy are presented in Figure 6b. The stored energy was kept below the upper bound, ensuring the passivity of the system. These results indicate that the proposed method can provide stable and realistic haptic feedback for teleoperation tasks involving unknown environments and varying contact conditions.

### 3.3. Human Subject Experiments

#### 3.3.1. Participant Recruitment

A total of 21 participants (13 males, 8 females; age range 21–34 years) from different backgrounds were recruited. They were all right-handed. None of them had previous experience with haptic feedback or human–robot interaction. No one reported any deficiencies in perception abilities. Furthermore, they were all naive to the purpose before the experiment. The recruiting process was supervised by the Institutional Review Board (IRB) of the Ethics Committee of Tongji Medical College, Huazhong University of Science and Technology with permission No.IORG0003571.

#### 3.3.2. Experimental Conditions

The main objective of this experiment was to validate that the proposed method could provide intuitive and compliant remote sensing for the operator. To fulfill this objective, the participants were required to teleoperate a robot manipulator to probe different objects and distinguish the types of objects through the force feedback mechanisms. Two kinds of silicone phantom and a book (see Figure 4b) were used to represent three types of tissue with different levels of stiffness; namely, soft, medium, and hard.

The experiment was conducted with two conditions: one with the proposed stiffness-matching force feedback mechanism, and the other one with the baseline method utilizing an energy tank-based direct force feedback mechanism derived from [25]. The force feedback controller of the baseline can be written as

(36) $$f_{m}^{\prime }\left(t\right)=-f_{e}\left(t\right)-D_{m}^{\prime }{\dot{x}}_{m}\left(t\right)$$

The baseline conveyed contact force directly for remote sensing and injects damping $D_{m}^{\prime }$ to make the system passive. The injected damping and the upper bound of the energy tank adopted in the baseline were set as $D_{m}^{\prime }=50$ Ns/m and $\bar{T}=20$ J, respectively.

#### 3.3.3. Procedure

Upon arrival at the experimental site, the participants were required to complete the consent form. Then, the staff demonstrated to the participants how to operate the teleoperation system while informing them of the experimental procedures. Before the experiment, each participant had a 5-minute practice time to become familiar with the teleoperation system. The participants were allowed to perceive the stiffness of these objects intuitively through the teleoperated robot during the practice procedure. They could probe the objects they wanted to perceive and needed to remember differences among the perceived stiffness through the master device. When the experiment started, they identified the probed object based on the perception in the practice procedure.

During the experiment, the participants controlled the slave robot to probe the target objects by manipulating the master device. There was a curtain between the master device and the slave robot, which prevented the participants from directly observing the slave side. The subjects were required to distinguish the type of objects through the feedback force.

Each participant completed 12 trials in each condition. Before each trial, the staff randomly selected a type of object for the subject to probe. The total number of trials interacting with each type of object within an experiment was equal. After the participants executed a trial, they were asked to fill out a questionnaire in Table 1 to investigate their identification of the type of the object they probed and their subjective evaluation of the stability, flexibility, and user satisfaction with the performance of the different conditions. Then an experiment trial was completed.

#### 3.3.4. Metrics

After collecting the questionnaire results and the physical data during the experiment, several metrics are proposed to evaluate the performance of the probing tasks. The accuracy of the perceived object answered by each participant is measured to validate the remote sensing and is expressed as follows

(37) $$A=\frac{\sum_{i=1}^{N}c\left(i\right)}{N}$$

where c(i)=1 if the participant recognized the remote object correctly, otherwise c(i)=0. N is the total number of trials per participant and has been given previously.

The stability is a subjective metric to describe the magnitude of the recoiling delivered to the participant by the master device during the participant’s teleoperation probing. Participants subjectively rated stability according to the magnitude of perceived recoiling. Then, the strenuousness of the induced motions produced by the two methods being tested were compared based on the participants’ ratings of stability.

The flexibility is a subjective metric used to investigate whether the injected damping in Equations (28) and (36) causes the operator to feel discomfort during the human–robot interaction, since damping may cause resistance to move the master device. Participants rated the flexibility by the smoothness they felt while operating the master manipulator. Then, the operator’s preference for the amount of damping injected was analyzed based on the participants’ ratings of flexibility.

The satisfaction is used to evaluate participants’ preferences for the two tested methods. Operators gave subjective scores on satisfaction after all trials were completed.

The average scores of the participants’ ratings for stability, flexibility, and satisfaction are calculated to evaluate the system performance of the two methods.

The total power flows through the human–robot interface during the identification process is calculated with the following

(38) $$H=\frac{\sum_{i=1}^{K}\left|f_{m}\left(i\right)\left(x_{m}\left(i\right)-x_{m}\left(i-1\right)\right)\right|}{K/h}$$

where *K* represents the sampling length of each probing process and *h* is the sampling frequency which is set as 50 Hz. The sum displacement of the participant’s hand during the perception process is recorded to characterize the human hand motion.

#### 3.3.5. Hypotheses

**Hypothesis** **4.** *Compared to the baseline, the proposed adaptive force feedback mechanism can improve the accuracy of operators in identifying remote objects.*

**Hypothesis** **5.** *The developed haptic interface enables a more stable interaction between the user and the master device.*

**Hypothesis** **6.** *Excessive damping injection can deteriorate the comfort of teleoperation.*

#### 3.3.6. Subjective Results

**Figure 7:**
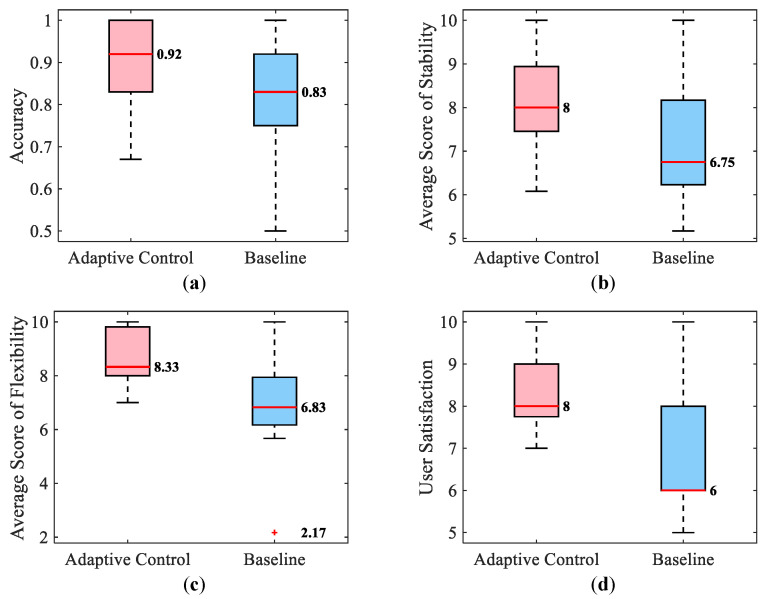
Subjective results of the human subject experiments. The red line represents the median of the data. Furthermore, the red plus symbol denotes an outlier in the data. (**a**) Accuracy of the classification. (**b**) Scores of contact stability. (**c**) Scores of flexibility. (**d**) Scores of user satisfaction.

The statistical results for the four subjective metrics are shown in Figure 7. The box plots in Figure 7a illustrate that the accuracy of distinguishing the mechanical characteristics of the environment with the adaptive stiffness teleoperation method performed better than the baseline method with the energy tank mechanism (supporting Hypothesis 4). Figure 7b–d present participants’ evaluations of the trials in box plots. The stability score describes how stable and smooth the probing contact process is, with a high score indicating less motion induced in the subject (supporting Hypothesis 5). The flexibility scores indicate that participants thought a small amount of damping injection resulted in a more comfortable operating feeling (supporting Hypothesis 6). To ensure the passivity of the system, the baseline needs to inject more damping than the proposed method, which destroys the comfort of teleoperation. Finally, the user satisfaction score provides an overall evaluation of the user experience offered by the teleoperation system.

Student *t*-tests were conducted to verify whether there was a significant difference between the proposed approach and the baseline, whose results were summarized in Table 2. Based on the results shown in Figure 7 and Table 2, it can be concluded that the proposed teleoperation can provide a more smooth, dexterous, and intuitive user experience with a more accurate perception of the mechanical property of the interacted material on the slave side, compared to the baseline method.

#### 3.3.7. Quantitative Results

**Figure 8:**
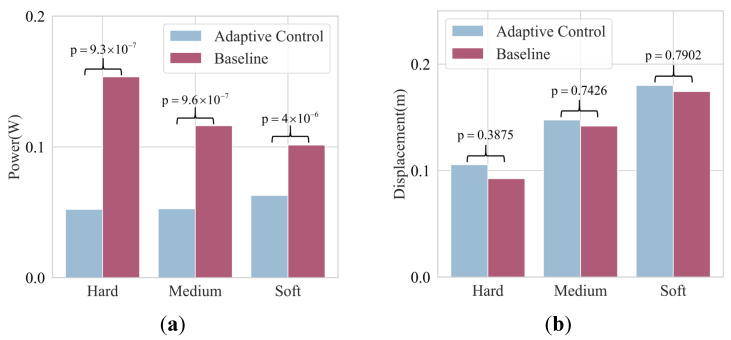
The mean values of total power and human hand displacement as well as the results of the Student’s *t*-tests. (**a**) The average powers when participants interact with different objects. (**b**) The average human hand displacements when participants interact with different objects.

The results in Figure 8a show that the proposed adaptive control generates lower power flow during probing compared to the energy tank-based direct force feedback control across all types of objects. This implies that the operator can perform the perception task more easily using the proposed approach and that the passivity of the system is better maintained due to lower power flows through the human–robot interface. Significant differences in power flow between the two methods are indicated by the results of the Student’s *t*-tests. Notably, Figure 8b shows that participants make slightly more hand displacements when using adaptive control. This is due to participants’ autonomous reciprocal motion during probing. However, participants become more cautious when they feel the recoiling and eventually decrease hand displacement. The results of the Student’s *t*-tests in Figure 8b show that there is no significant difference in human hand motion between the two methods.

In summary, the proposed method produces less power flow with higher identification accuracy and similar total human hand displacements. This is evidenced by Figure 7a and Figure 8.

## 4. Discussion

The transparency of a teleoperation system, referring to how well the complete system can convey the environment to the user, was believed to be achieved by minimizing the differences between the external force on the slave side and the feedback force on the master side [11]. Following this idea, complex bilateral teleoperation frameworks have been developed to directly realize the exact interaction force on the master device [25,28]. In this study, an interesting finding contradicted this idea. The accumulated errors between feedback forces and contact forces when using the proposed adaptive control and the baseline are measured and the means of these errors are calculated as shown in Table 3. It is clear that the proposed teleoperation method had greater force-tracking errors than the baseline method.

**Figure 9:**
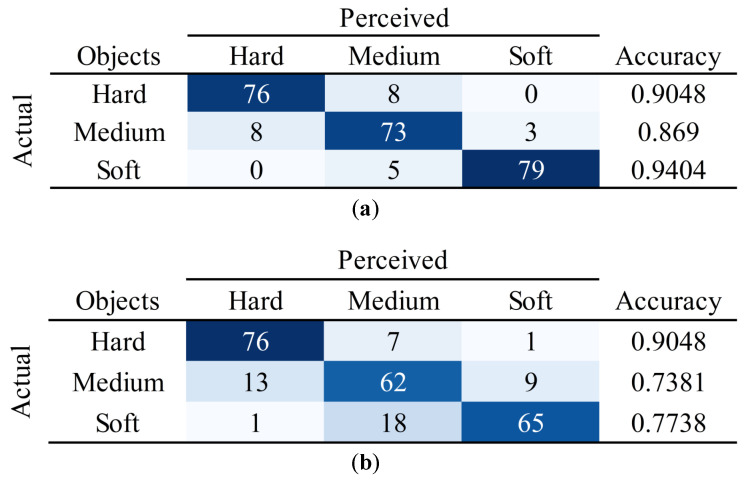
Confusion matrices of two methods. (**a**) The confusion matrix of the adaptive force feedback control (the proposed method). (**b**) The confusion matrix of the energy tank-based direct force feedback control (the baseline).

The identification accuracy of participants for each object when using both methods was counted in the confusion matrices plotted in Figure 9. It can be seen that when probing the hard object, both methods enable participants to obtain a high perceptual accuracy. Furthermore, when probing objects with medium and soft stiffness, participants identify remote objects more precisely with the adaptive method. Therefore, it can be empirically concluded that the adaptive force feedback significantly helps the participants to identify the objects. From Table 3 and Figure 9, it can be concluded that when designing a force feedback controller for a bilateral teleoperation system, it is necessary to convey stiffness that characterizes the mechanical properties of remote objects rather than directly convey the external force through the haptic feedback interface.

This study is inspired by the phenomenon that when a human contacts an unknown object directly, he or she can quickly adjust the hand stiffness to adapt to the environment. It is assumed that the motion of the human hand collected by the master device reflected the desired position the operator intends to achieve. The corresponding hand stiffness is estimated based on the interactive force measured on the slave side. The objective of this process is to reproduce the direct interaction between the operator and the environment on the master side. So the operator can adjust the hand stiffness in time to achieve stable contact and intuitively perceive the environmental characteristics. From the results of the human subject experiment, we empirically verified that the estimated human hand stiffness can indirectly describe the mechanical properties of remote objects.

We acknowledge a limitation of the proposed method in practical teleoperation. The proposed method might overly simplify the human haptic adaptation with an RLS process. Though the effectiveness of the proposed method was verified empirically through human subject experiments, a rather complex human haptic learning model could contribute to a more intelligent teleoperation framework that could help people fulfill more complicated tasks remotely. Wearable devices can collect various physiological and physical signals from the human body through biosensors. As described in [37], the collected signals can be processed to calculate the required parameters of the human body. Electromyography (EMG) signals can be obtained by monitoring muscle activity and have been used to estimate body impedance parameters [38]. Consequently, EMG signals will be considered for estimating human hand stiffness in future studies. The fuzzy approximation method [39] will be regarded for compensating the nonlinear terms in the signal processing process.

## 5. Conclusions

This paper proposes a control mechanism for teleoperated surgical robotics that takes into account human adaptability, which enhances the operator’s intuitive perception of remote objects. The passivity is maintained through the two-layer control structure with an energy tank. The results of stable contact tests and human subject experiments empirically verify that the proposed method can ensure stable contact and enable the operator to perceive the stiffness of remote objects more accurately, which shows the promise of considering human adaptability in the design of force feedback controllers.

In the future, we plan to measure the EMG signals of human arm muscles during haptic interactions. Through investigating the EMG signals, the nature of human haptic adaptation could be captured with sophisticated models, which we believe could help us achieve complicated physical HRI to fulfill remote operation tasks.

## Figures and Tables

**Table 1:** Questionnaire of the human subject experiments for subjective metrics.

Question	
Accuracy	Which object do you think is on the slave side?
Stability	How would you rate the stability of the system from 0 to 10 according to the degree of recoiling that you felt during the trial?
Flexibility	How would you rate the flexibility of the system from 0 to 10 according to the smoothness of moving the master device during the trial?
Satisfaction	How would you rate your satisfaction with the system from 0 to 10?

**Table 2:** Total average values and results of *t*-tests.

Metric	Adaptive Control	Baseline	*p*-Value
Accuracy	0.901	0.801	0.0136
Contact Stability	8.194	7.196	0.0153
Flexibility	8.681	6.953	$4.5734\times 10^{-4}$
Satisfaction	8.190	6.953	0.0014

**Table 3:** Means of accumulated force errors when participants probe different objects.

	Adaptive Control	Baseline
Hard	2.601	0.6409
Medium	2.9447	0.1543
Soft	1.415	0.1194

## Data Availability

Currently, the datasets generated and analyzed during the current study cannot be made publicly accessible due to privacy protection.

## Abbreviations

The following abbreviations are used in this manuscript:

RLS	Recursive Least Square
TDPA	Time Domain Passivity Approach
ROS	Robot Operating System
